# Supplementary material for: Modelling of onchocerciasis-associated skin and ocular disease and the impact of ivermectin treatment
Source: Commun Med (Lond). 2026 Mar 2;6:198. doi: 10.1038/s43856-026-01464-2 (PMC13066558; doi:10.1038/s43856-026-01464-2)
Supplement: Supplementary file 2 — Supplementary Information [file 43856_2026_1464_MOESM2_ESM.pdf]

Supplementary Information: Modelling of onchocerciasis-associated skin and ocular disease and the impact of ivermectin treatment

Matthew A. Dixon[1†*], Aditya Ramani[1,2†], Martin Walker[1,2], Jacob N. Stapley[1], Michele E. Murdoch[3], Ian E. Murdoch[4], Gladys A. Ozoh[5], Jonathan F. Mosser[6,7], Maria-Gloria Basáñez[1*]

[1]MRC Centre for Global Infectious Disease Analysis and London Centre for Neglected Tropical Disease Research, Department of Infectious Disease Epidemiology, School of Public Health, Imperial College London, 90 Wood Lane, London, W12 0BZ, UK

[2]Department of Pathobiology and Population Sciences, Royal Veterinary College, Hawkshead Lane, Hatfield, Hertfordshire, AL9 7TA, UK

[3]Department of Dermatology, West Herts Teaching Hospitals NHS Trust, Watford, Herts., UK

[4]International Centre for Eye Health, Institute of Ophthalmology, London, UK

[5]Dermatology Division, University of Nigeria Teaching Hospital, Ituku Ozala Enugu State, Nigeria

[6]Institute for Health Metrics and Evaluation, Hans Rosling Center for Population Health, 3980 15th Ave NE, Seattle WA 98195, USA

[7]Department of Global Health, School of Public Health, and Seattle Children's Hospital, University of Washington, Seattle WA, USA

[†]Contributed equally

[*]Corresponding authors: m.dixon15@imperial.ac.uk and m.basanez@imperial.ac.uk

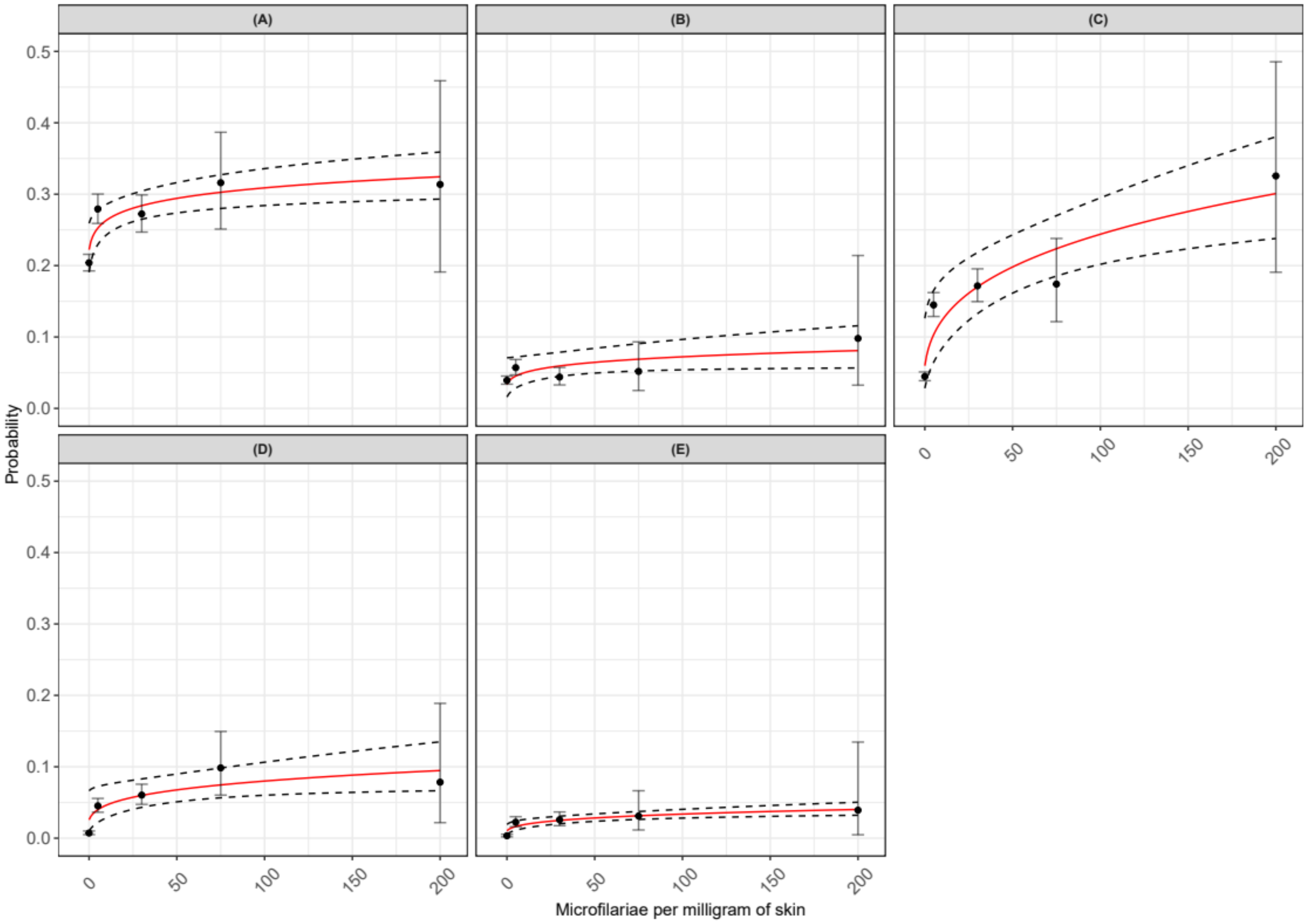

Supplementary Fig. 1. Probabilities of developing onchocerciasis skin disease (OSD) sequelae as a function of microfilarial load. Black circles are estimates from Murdoch *et al*.[1] with Clopper-Pearson 95% confidence intervals[2]; red lines represent a generalized linear model (GLM) with a log-link function fitted to the data using R v.4.3.2 (https://cran.r-project.org/bin/windows/base/); dashed lines are the 95% confidence intervals around model predictions. The GLM is $Y(M_{(i)}) = \exp\{\beta_1[\log(M_{(i)}+1)] + \beta_0\}$ where $Y(M_{(i)})$ represents the probability of developing each OSD sequela as a function of $M_{(i)}$, the individual's microfilarial load (mean no. of mf/mg) from two iliac crest snips taken from each individual; $M_{(i)}$ is taken as the mid-point of the binned mean microfilarial load according to the bins (0 to <1, 1 to <10, 10 to <50, 50 to <100, ≥100) presented in the Supplementary File S1 of Murdoch *et al*.[1]; β_0 is the intercept, such that a microfilarial load of zero can be associated with a non-zero OSD probability (to account for sampling error in the skin-snipping process), and β_1 is the strength of the association between microfilarial load and the probability of developing OSD. **A** Severe itch (β_0 = –1.504; β_1 = 0.071). **B** Reactive skin disease (β_0 = –3.390; β_1 = 0.165). **C** Atrophy (β_0 = –2.817; β_1 = 0.305). **D** Depigmentation (β_0 = –3.659; β_1 = 0.246). **E** Hanging groin (β_0 = –4.571; β_1 = 0.256).

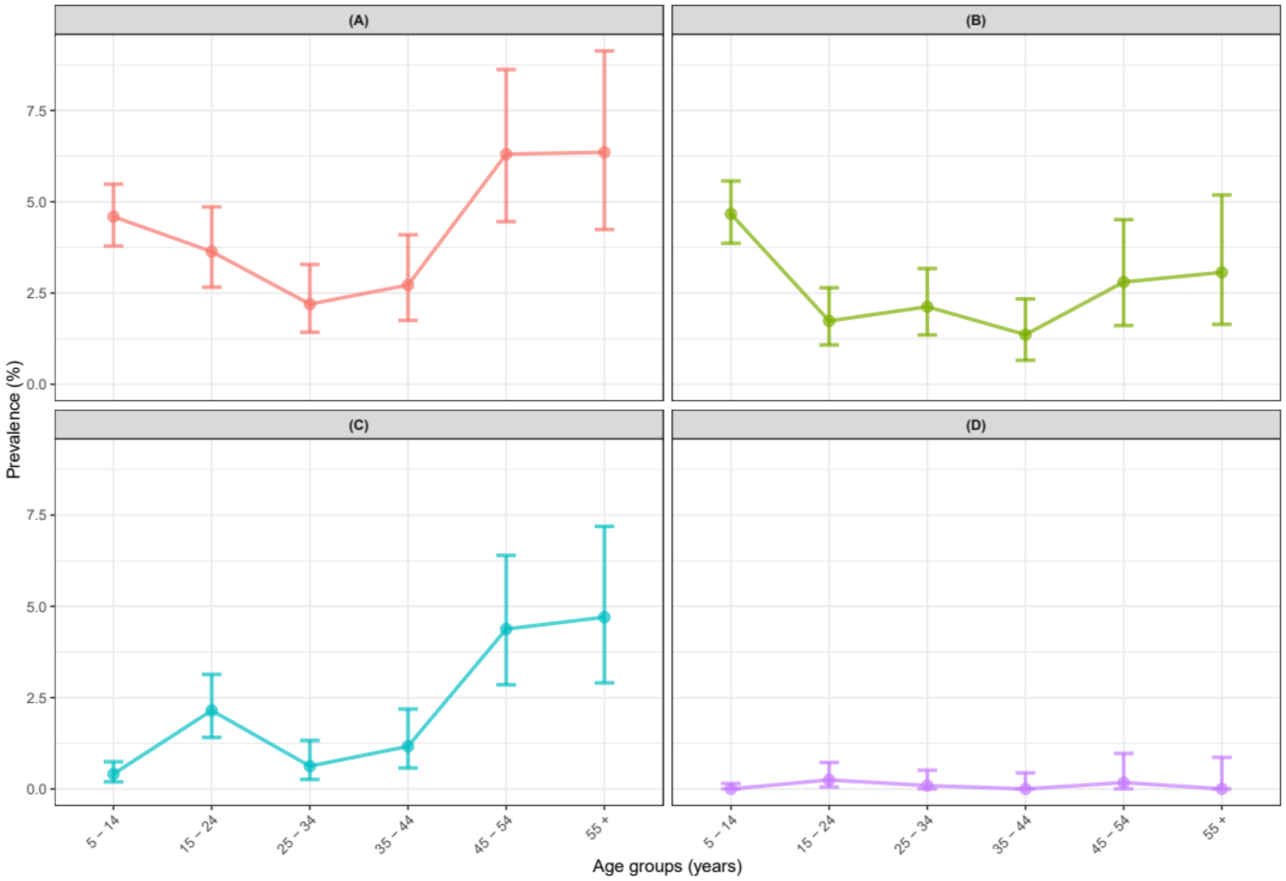

Supplementary Fig. 2. Age-prevalence profiles for the reversible onchocerciasis skin disease sequelae within the reactive skin disease category. Circles are prevalence estimates from Murdoch *et al.*[1] with Clopper-Pearson 95% confidence intervals[2]. **A** RSD: Reactive skin disease. **B** APOD: Acute papular onchodermatitis. **C** CPOD: Chronic papular onchodermatitis. **D** LOD: Lichenified onchodermatitis.

Supplementary Text 1. Further EPIONCHO-IBM details

Exposure heterogeneity and density dependence

The stochastic, individual-based EPIONCHO-IBM[3] has been developed from its deterministic, population-based (EPIONCHO) predecessors[4-7]. The model tracks, in a closed population (of 2,000 individuals for this work), the number of male and (fertile/non-fertile) female *Onchocerca volvulus* adult worms in human hosts (modelled using stochastic difference equations); the number of skin microfilariae (per mg of skin or per skin snip, modelled deterministically by using a partial differential equation that accounts for the contribution of each age class of adult female worms to the number of microfilariae in individuals assuming a worm's age-specific fecundity rate), and the number of infective, L3 larvae in blackfly vectors (modelled deterministically). EPIONCHO-IBM generates a 'true' microfilarial load and an 'observed' microfilarial load (taking into account skin-snip sensitivity)[3]. Parasite population abundance is regulated in humans and flies by density-dependent processes operating upon establishment of incoming worms within humans; establishment of L3 larvae within vectors, and vector survival[5]. Excess mortality of humans as a function of their microfilarial load[8,9] has not yet been included. For sub-Saharan Africa settings, the model has been parameterised for savannah *O. volvulus–S. damnosum* sensu lato (s.l.)[3,5,6]. The baseline (pre-control) microfilarial prevalence (indicative of the endemicity level) is determined by the annual biting rate (ABR, no. bites/person/year)[3,6]. Unless vector control is explicitly modelled, the transmission conditions are assumed to remain constant throughout the simulations in the absence of robust data to indicate changes in vector density or secular trends due to environmental change.

Individuals within the model are differentially exposed to blackfly bites depending on their age and sex[4,10] as well as on their individual specific exposure, $E_{(i)}$. This individual exposure factor is assigned at birth and drawn from a gamma distribution,

$$E_{(i)} \sim G(k_E, \beta_E) \qquad \text{Eqn. (S1)}$$

where k_E is the shape parameter and β_E the rate parameter. It is assumed that $k_E = \beta_E$, such that the mean exposure in the population is unity, i.e., blackfly bites are distributed among hosts with an average exposure given by the ABR[3]. The k_E parameter defines the degree of inter-individual exposure heterogeneity, whereby lower values of k_E represent stronger overdispersion in exposure heterogeneity and vice-versa. In this work we used k_E values equal to 0.3 or 0.4[3].

Parasite population regulation is assumed to depend on parasite density. Density-dependent processes are important in helminth transmission dynamics, as they contribute to parasite

population stability and resilience to interventions[11,12]. In EPIONCHO-IBM, density dependence is assumed to operate upon the establishment and development to the infective L3 stage of ingested microfilariae within blackflies, whose survival is also affected by the density of microfilarial intake (a function of the intensity of microfilaridermia in the human host)[5]. Parasite establishment within humans is assumed to depend on transmission intensity, measured by the annual transmission potential (ATP, the no. of L3 larvae potentially received per person per year, which in the model is a function of the ABR and the mean no. of L3 per blackfly)[3,5,6,13]. Different values of k_E are associated with different values of the parameters describing the establishment and development to adult worms of the L3 larvae transmitted from vectors to humans, as shown in Supplementary Table 1.

Supplementary Table 1. Shape (equal to rate) parameter of the gamma distribution of individual exposure and associated density dependence parameters in EPIONCHO-IBM.

Shape parameter of gamma distribution	Density dependence parameters for parasite establishment within humans		
k_E	δ_{H_0}	δ_{H_∞}	c_H
0.2	0.385	0.003	0.008
0.3	0.186	0.003	0.005
0.4	0.118	0.002	0.004

Where δ_{H_0} is the proportion of L3 larvae developing to adult worms within the human host, per bite, when ATP tends to 0; δ_{H_∞} is the proportion of L3 larvae developing to adult worms within the human host, per bite, when ATP is very large, and c_H is the severity of transmission intensity-dependent parasite establishment within humans. A full description of EPIONCHO-IBM can be found in Hamley *et al.*[3]

Treatment coverage and proportion of never-treated population

We used the controlled treatment correlation approach proposed by Dyson *et al.*[14], whereby each individual in the population is assigned a probability of attending any round, drawn from a Beta distribution. The correlation parameter (ρ) controls the magnitude of correlation between attendance to consecutive treatment rounds by individuals and is related to the Beta distribution α and β parameters following the expressions:

$\alpha = c(1-\rho)/\rho$ and $\beta = (1-c)(1-\rho)/\rho$ Eqns. (S2, S3)

where c is the coverage of total population (Matthew Graham, pers. comm.). A ρ value of 0 indicates no correlation between the treatment rounds attended by an eligible individual (with eligibility assigned according to individuals' age; children under the age of 5 years do not receive treatment), such that individuals are randomly assigned to receiving treatment at each

round. (NB when ρ = 0 we cannot divide by zero, so the model assumes that treatment is random with coverage c.) A ρ value of 1 indicates a fully systematic scheme such that those individuals which are assigned to receiving treatment in round 1 will always receive treatment in subsequent rounds. Specifying ρ values between 0 and 1 allows for a lesser or greater degree of systematic treatment adherence in the simulated treatment programme. In addition, a fixed proportion of the eligible population was randomly assigned to never receiving treatment, which was required to attain the never-treated proportion reported after 5 or 6 treatment rounds in the study sites of Taraba (Nigeria) and Bushenyi (Uganda)[15] (see Table 2 of Main Text and Supplementary Fig. 5).

Supplementary Text 2. Integration of onchocerciasis skin disease sequelae (OSD) into EPIONCHO-IBM

At every daily time-step in the model, individuals are at risk of developing OSD sequelae if the criteria of being currently sequela-negative and microfilaria (mf)-positive are satisfied (Fig. 1 in Main Text). For the reversible and irreversible OSD sequelae, we assumed that individuals under the age of two years would not be able to develop such sequelae. Since the pre-patent period of *Onchocerca volvulus* is 2-3 years[16], we assumed that individuals needed to be at least 2 years old before they could become OSD sequela-positive. Those individuals at risk of developing OSD sequelae undergo a Bernoulli trial with (daily) probabilities of developing each sequela given in Table 1 of the Main Text. For the reversible conditions, individuals revert to being sequela-negative after a duration of 3 days which, after testing periods between 1 and 5 days, was the most consistent with the age-prevalence OSD profiles presented in Fig. 3 (Main Text). Individuals remain sequela-positive for the irreversible conditions.

For both reversible and irreversible sequelae, we ignore any age-dependent variation in exposure and/or susceptibility to developing disease, such as increasing risk from past infection or changing susceptibility with age.

For reversible OSD, we assumed that sequelae would be sufficiently transient that prevalence (after subtracting background morbidity) reflects underlying incidence and that any accumulation of past morbidity is negligible.

For irreversible OSD, we assumed that infected (microfilaria-positive) individuals have experienced a daily risk of developing the sequelae for a period equal to the average age of the sampled population (which was 25 years in the dataset we used[1]), subtracting the first 2 years of life, when it is assumed that morbidity cannot develop (Eqn. (1) in Main Text).

Supplementary Text 3. Integration of onchocerciasis ocular disease sequelae (OOD) into EPIONCHO-IBM

At every daily time-step in the model, individuals are at risk of developing blindness if the criteria of being not currently blind or not assigned to become blind in the next two years, and having a non-zero 'true' microfilarial count are satisfied. We assume a delay of 2 years between being at risk of developing blindness and harbouring a given microfilarial load, such that microfilarial load 2 years in the past, and not current microfilarial load determines blindness risk[17]. In Eqn. (2) of the Main Text, adapted from Little *et al*.[17], the probability of blindness onset is related to an individual's measurable (by skin snip) microfilarial count. However, in the model we identified individuals at risk of developing blindness based on their 'true' microfilarial count, rather than their count according to detectable microfilariae by skin-snip microscopy (see Supplementary Text 1, Exposure heterogeneity and density dependence), enabling the model to generate small blindness probabilities in those individuals with false negative microfilarial measurements. Those individuals at risk of developing blindness undergo a Bernoulli trial to determine whether they will become blind (2 years later).

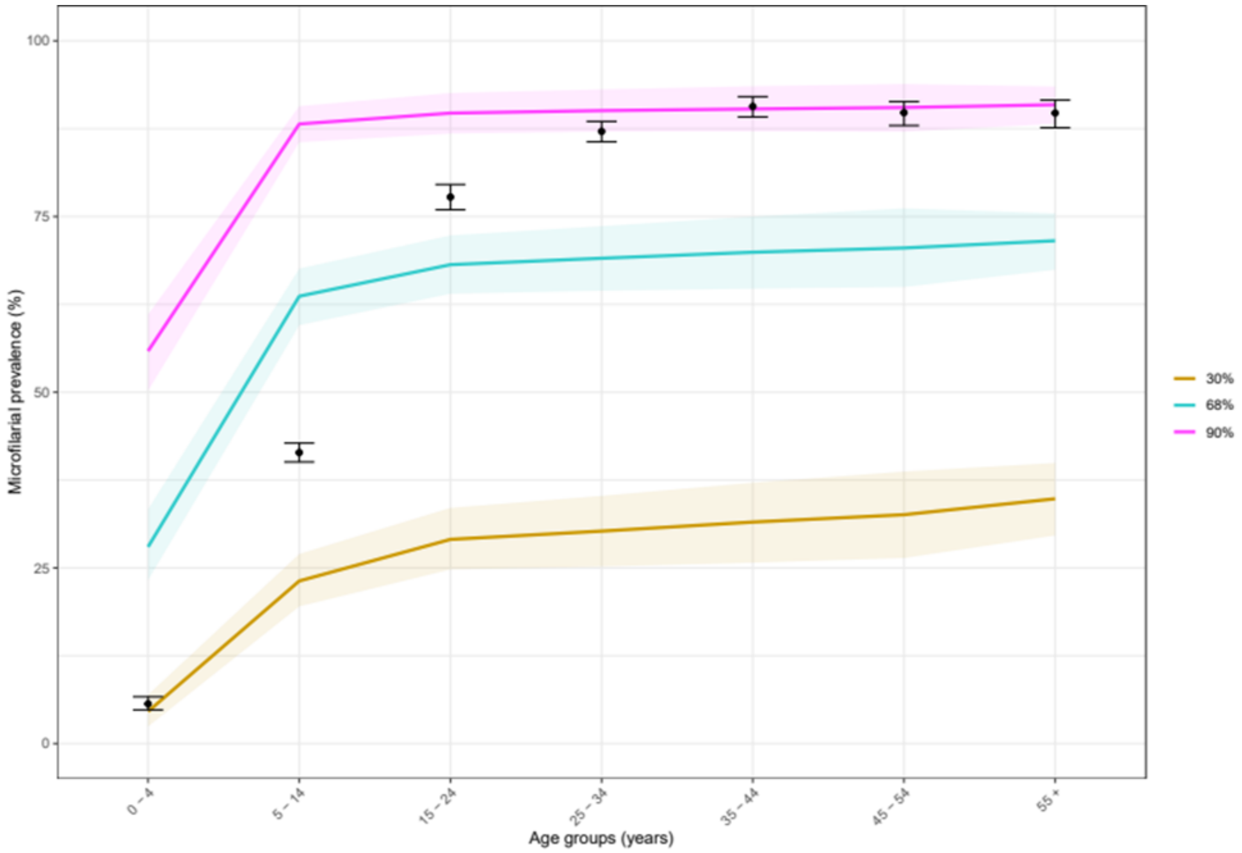

Supplementary Fig. 3. Age-prevalence profiles of *Onchocerca volvulus* skin microfilariae in the Onchocerciasis Control Programme in West Africa. Age-prevalence profiles were generated using EPIONCHO-IBM with annual biting rate (ABR) of 285, 2,000 and 60,000 bites/person/year and individual exposure parameter k_E= 0.3 for, respectively, microfilarial prevalence of 30%, 68% and 90% for data from Kirkwood *et al*.[18]. Solid lines are the means of 1,000 model runs; shaded areas are the 95% uncertainty intervals (2.5th to 97.5th quantiles of stochastic model predictions); black circles are prevalence estimates with Clopper-Pearson 95% confidence intervals[2].

Supplementary Text 4. Converting nodule prevalence into microfilarial prevalence

We used the procedure described by Coffeng (2024) in the Zenodo repository https://zenodo.org/records/13969100[19]. This repository provides a set of posterior distribution draws (posterior_sample.csv) as well as detailed instructions for their use (posterior_sample_instructions.docx) to convert onchocercal nodule prevalence in adult males (aged ≥20 years) into microfilarial prevalence in the general population (aged ≥5 years). The posterior draws are based on the analysis of (paired) field data on prevalence of nodules and skin microfilariae from onchocerciasis-endemic villages presented in Coffeng *et al*. (2013)[20],

and in particular on the detailed description of the statistical model given in Supplementary S1 Text[20]. Briefly, the conditional distribution of village-level microfilarial prevalence given nodule prevalence is formulated using (univariate or multivariate) normal distributions for the logit-transformed prevalences (parameterised in terms of mean and variance or covariance). The observed, ‘apparent’ nodule prevalence is corrected according to the diagnostic performance parameters of nodule palpation to provide ‘true’ nodule prevalence. The comma-separated “posterior_sample.csv” file[19] contains a large sample of draws from the joint posterior distribution of the vector of overall mean microfilarial prevalence and nodule prevalence and its covariance, village-level standard deviation of microfilarial and nodule prevalence and their correlation, and specificity of nodule palpation. An algorithm is provided in the “posterior_sample_instructions.docx” file to generate a posterior predictive draw for microfilarial prevalence, conditional on a posterior draw of nodule prevalence[19].

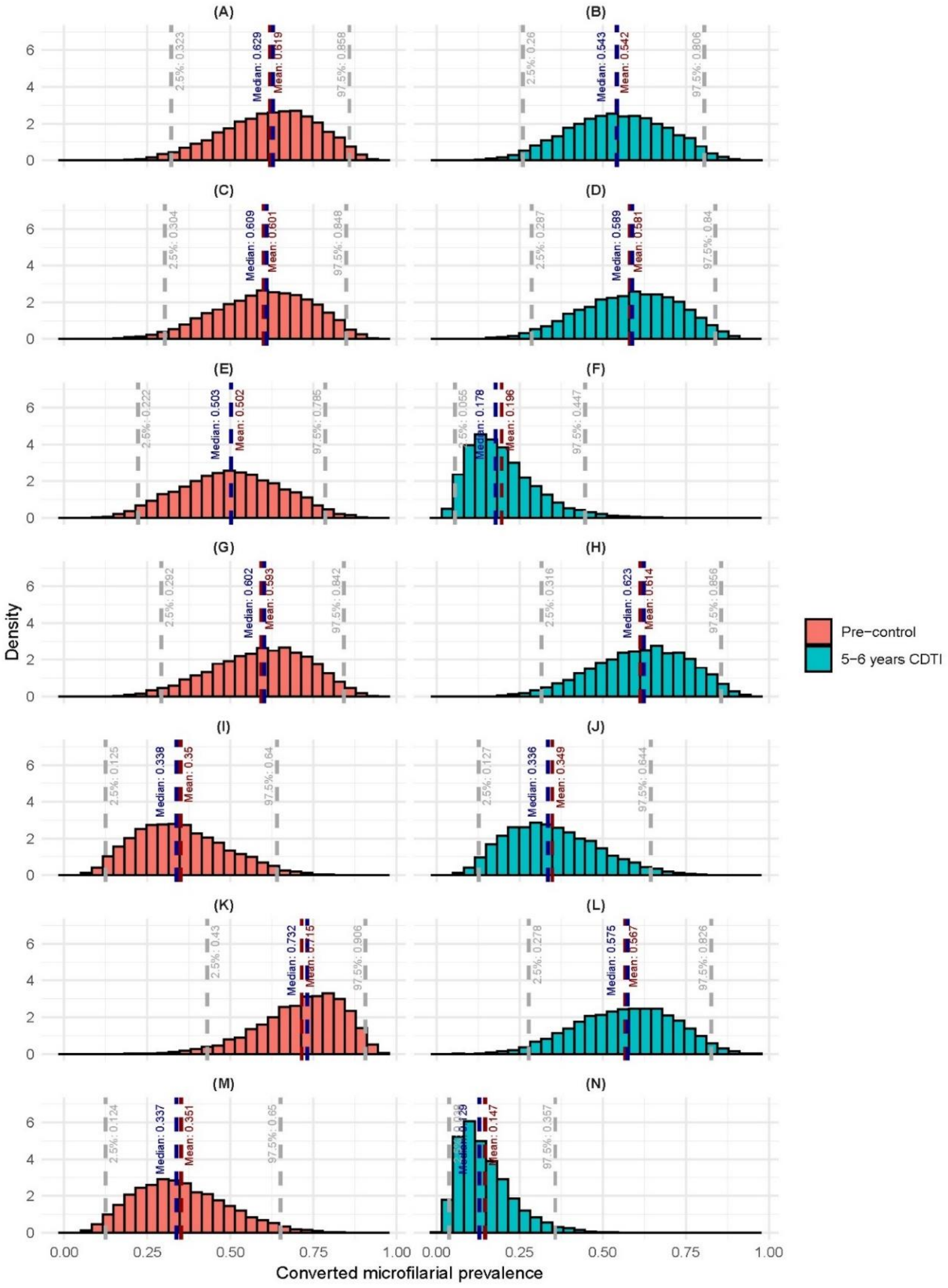

Supplementary Fig. 4. Posterior distributions of converted microfilarial prevalence from nodule prevalence. Distributions were generated from nodule prevalence estimates collected before the implementation of community-directed treatment with ivermectin (CDTI) (pre-control) and 5-6 years into the CDTI programme in seven sites across Cameroon: **A**, **B** Kumba (rainforest). **C**, **D** Ngambe (forest-savannah mosaic). Nigeria: **E**, **F** Cross River (rainforest). **G**, **H** Kogi (forest-savannah mosaic). **I**, **J** Taraba (savannah). Sudan: **K**, **L** Raja (savannah). Uganda: **M**, **N** Bushenyi (rainforest). Data are from Ozoh *et al.*[15]. The conversion of nodule into microfilarial prevalence was conducted according to Coffeng[19] and Coffeng *et al*.[20] (see Supplementary Text 4). Light grey dashed lines indicate 95% credible intervals; dark blue dashed lines correspond to the medians, and dark red dashed lines indicate the means of the distributions. The median values of microfilarial prevalence were used to determine the annual biting rates (ABR, no. bites/person/year) necessary to simulate the baseline epidemiological conditions in the seven study sites (Table 2 of the Main Text).

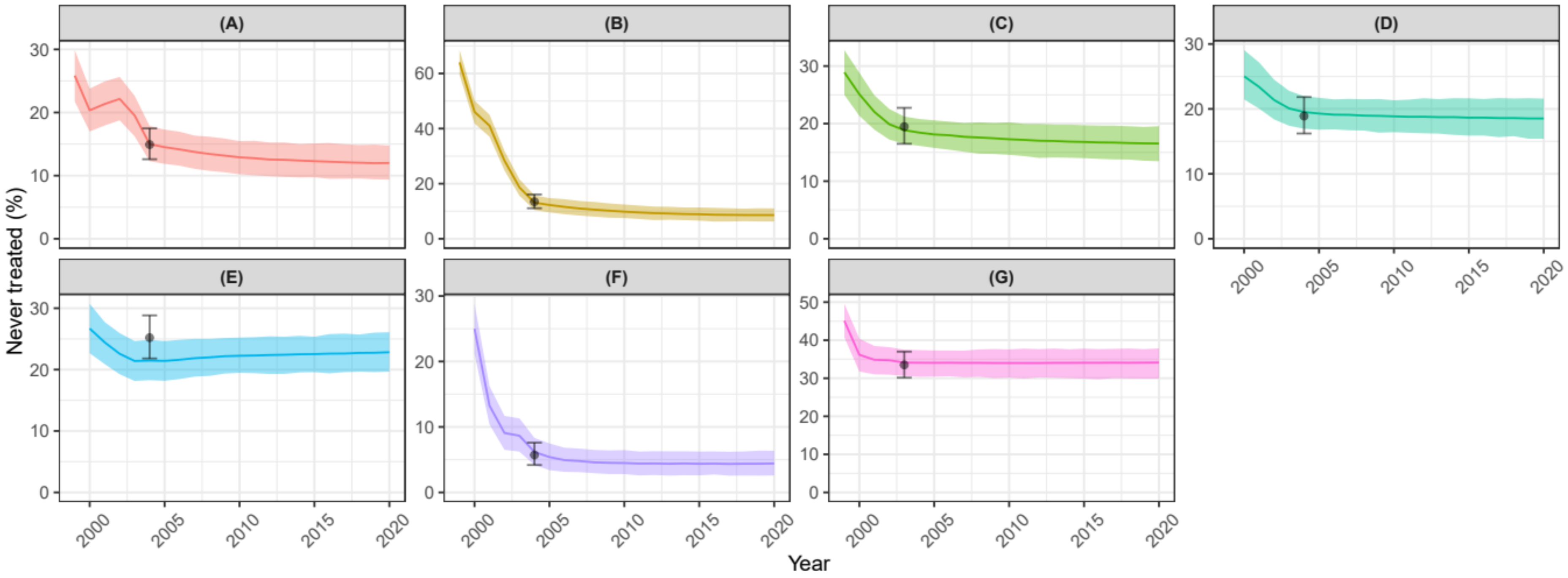

Supplementary Fig. 5. Modelled proportions (in percent) of never-treated population compared to data across seven study sites. Cameroon: **A** Kumba. **B** Ngambe. Nigeria: **C** Cross River. **D** Kogi. **E** Taraba. Sudan: **F** Raja. Uganda: **G** Bushenyi. The trajectories of the modelled proportions of never-treated population are based on the estimations of the correlation parameter ρ as described in Supplementary Text S1 (Treatment coverage and proportion of never-treated population) to match the data presented by Ozoh *et al*.[15] in the seven study sites 5 or 6 years into community-directed treatment with ivermectin (CDTI) (see Table 2 of the Main Text). Solid lines are the means of 1,000 model runs; shaded areas are the 95% uncertainty intervals (2.5th to 97.5th quantiles of stochastic model predictions); black circles are estimates with Clopper-Pearson 95% confidence intervals. These results were used to model the impact of CDTI on onchocerciasis skin disease (OSD) presented in Figs. 5-8 of the Main Text. NB: *y*-axis in different scales.

Supplementary Text 5. Modelling for policy: PRIME-NTD

We adhered to the Five Principles of the Neglected Tropical Diseases (NTD) Modelling Consortium for good practice in policy-relevant NTD modelling[21] when conducting this research. Supplementary Table 2 briefly describes the five principles, how they were fulfilled, and where these principles are addressed in the Main Text and/or Supplementary Information.

Supplementary Table 2. Policy-Relevant Items for Reporting Models in Epidemiology of Neglected Tropical Diseases (PRIME-NTD) summary table[21].

Principle	What has been done to satisfy the principle?	Where in the manuscript is this described?
**Stakeholder engagement**	Discussions with a range of collaborators, as well as experts in onchocerciasis dermatology and ophthalmology.	Author list, Acknowledgements section.
**Complete model documentation**	References to the full model description of EPIONCHO-IBM are provided. An Open Access link to the code is provided.	Methods section, Supplementary Information and Code availability section.
**Complete description of data used**	All the data used are published and detailed in the manuscript, with cited references.	Main Text, Supplementary Information, Reference lists of Main Text and Supplementary Information.
**Communicating uncertainty**	95% confidence intervals around prevalence estimates are presented; 95% uncertainty intervals were calculated for 1,000 model repeats for all simulations.<br>To account for uncertainty in transmission settings across study areas, microfilarial and sequela age-prevalence profiles were generated for a range of annual biting rates.<br>For conversion of nodule prevalence into microfilarial prevalence, uncertainty in observed nodule prevalence was incorporated and 95% credible intervals are presented.	Methods and Results sections, including figures and figure captions in the Main Text and Supplementary Information.
**Testable model outcomes**	Modelled sequela age-prevalence profiles were compared to age-prevalence data. Model outcomes were tested against skin and ocular disease prevalence following annual ivermectin treatment in 9 settings.	Results and Discussion sections of Main Text. Supplementary Information.

Supplementary Text 6. Comparison of EPIONCHO-IBM and ONCHOSIM morbidity modelling approaches

We compare the salient features of each approach for modelling morbidity in EPIONCHO-IBM and ONCHOSIM in Supplementary Table 3. While it is beyond the scope for this analysis to compare their population biology and transmission dynamics features, a brief description of ONCHOSIM is provided. ONCHOSIM is an individual-based stochastic model of onchocerciasis transmission[5,22,23], simulating the life-histories of individual humans and *O. volvulus* adult worms within humans. The parasite's larval stages within blackflies are modelled deterministically. One major difference between EPIONCHO-IBM and ONCHOSIM is the assumption, in the former, of the operation of (negative) density dependence on the probability of parasite establishment within humans as a function of transmission intensity[13,24]. This, together with stronger exposure heterogeneity (see **Exposure heterogeneity and density dependence** in Supplementary Text 1), allows for low-prevalence settings to be endemically stable at low ABR values in EPIONCHO-IBM[25]. In contrast, an external force-of-infection process or an influx of infection from areas with higher endemicity (higher ABR values) is necessary to model low-prevalence dynamics in ONCHOSIM[26].

Supplementary Table 3. Comparison of structural and parametric assumptions for morbidity modelling in EPIONCHO-IBM and ONCHOSIM.

	EPIONCHO-IBM	ONCHOSIM
**Onchocerciasis skin disease (OSD)**		
Sequela modelled	Reversible: severe itch, Reactive skin disease (RSD).<br>Irreversible: atrophy (ATR), depigmentation (DPM), hanging groin (HG)	Reversible: severe itch, RSD, palpable nodules[27].<br>Irreversible: ATR, DPM, HG[27].
Link to parasite stage/process	Presence of microfilariae (mf) in individuals (being mf-positive).	Mortality of mf (severe itch, RSD, ATR, DPM, HG).<br>Number of adult female worms present in individual humans (nodules).
Time-step	Daily.	Monthly.
Structural approach to modelling sequelae	Individuals are at risk of developing sequela if satisfy criteria (Fig. 1 Main Text) at each time step. Those individuals at risk undergo a Bernoulli trial with (daily) probabilities (Table 1 Main Text) of developing each sequela. Those aged ≤2 years do not develop OSD.	Tissue damage mechanism: amount of damage is a function of i) individual's susceptibility to developing specific sequela (drawn from a gamma distribution); ii) accrued tissue damage is tracked; one or more thresholds (DPM can be mild or severe) reached per condition for sequelae to develop.

Process for capturing sequelae reversibility	Individuals revert to being sequela-negative (severe itch, RSD) after a duration of 3 days.	Regression rate included in function of amount of tissue damage for severe itch, RSD and nodules, with reversion occurring after tissue damage is reduced below threshold(s) for specific sequela.
Excess human mortality	Not included.	Not included.
Estimated/fitted parameters and datasets used	Daily probability of developing sequela estimated as the proportion of individuals presenting with the sequela who are mf-positive (after subtracting background morbidity) for reversible OSD, and estimated according to Eqn. (1) Main Text for irreversible OSD.<br>Data used for estimation of daily OSD probabilities from Murdoch *et al*. (2017)[1] for Kaduna State, northern Nigeria, savannah area; n = 6,643.	Individual susceptibility parameter fitted (shape and rate parameters $\alpha = \beta$ to ensure distribution mean = 1); disease threshold and regression rates.<br>Data used for fitting from Murdoch *et al*. (2002)[28] for Nigeria, Tanzania and Uganda. Data from Ghana and Cameroon omitted due to non-random sampling. The data subset used was considered to be mainly associated with forest areas; n = 4,810. Infection data in Murdoch *et al*. (2002)[28] was recorded as prevalence of palpable nodules, converted to microfilarial prevalence[19,20].
**Onchocerciasis ocular disease (OOD)**		
Sequela modelled	Irreversible: Visual impairment (VI), blindness.	Irreversible: VI, blindness[27].
Link to parasite stage/process	Microfilarial count in individuals lagged by 2 years (Eqn. 2 Main Text).	Mortality of mf in individuals.
Time-step	Daily.	Monthly.
Structural approach to modelling sequelae	Individuals are at risk of developing blindness if satisfy criteria (Fig. 2 Main Text) at each time step. When individuals are assigned to become blind after undergoing a Bernoulli trial, they do so 2 years later.	Tissue damage mechanism as for OSD. Two-stage disease progression, with a lower tissue damage threshold for VI compared to blindness. Lower VI threshold in savannah areas compared to forest areas.
Excess human mortality	Not included.	Reduction of an individual's residual life expectancy by 50% once they become blind[29].
Estimated/fitted parameters and datasets used	Probability of developing blindness according to Eqn. (2) Main text, following Little *et al*.[8].<br>This relationship was based on a pre-control cohort dataset collected during the Onchocerciasis Control	Individual susceptibility parameter fitted (shape and rate parameters $\alpha = \beta$ to ensure distribution mean = 1); disease thresholds fitted separately for savannah and forest areas.

	Programme in savannah areas; n = 297,756 individuals. VI prevalence calculated by multiplying blindness prevalence by a factor ranging from 0.5[30] to 1.78[31].	Pre-control data on community-level prevalence of infection and its relationship with prevalence of VI and/or blindness, from reported mf prevalence in those aged ≥5 years from savannah areas[32] and community microfilarial load data from forest[33-39] and forest-savannah mosaic areas[40].
**Onchocerciasis associated epilepsy (OAE)**		
Sequela modelled	Irreversible: OAE[41].	Irreversible: OAE[42].
Link to parasite stage/process	Mf count in individuals.	Mortality of mf in individuals.
Time-step	Daily	Monthly
Structural approach to modelling sequela	As described for irreversible OSD/OOD (selection criteria in Stapley *et al*. 2024[41], in the age range 3-15 years (not expanding to those aged up to 18 years, owing to lack of mf data to inform risk of OAE onset)[43,44].	Tissue damage mechanism as for OSD/OOD, modelled as brain damage. Once brain tissue damage threshold is exceeded, the individual develops OAE. Individuals older than 18 years do not develop OAE.
Excess human mortality	Not included.	Reduction of an individual's residual life expectancy once they develop OAE, randomly sampled from a uniform distribution.
Estimated/fitted parameters and datasets used	Daily probabilities of OAE onset estimated following Eqn. (1) of Stapley *et al*. (2024)[41]. Data from Mbam River Valley, Cameroon, forest-savannah mosaic area, n = 729[43].	Individual susceptibility parameter fitted (shape and rate parameters $\alpha = \beta$ to ensure distribution mean = 1); disease threshold and mean life expectancy reduction fitted.<br>Data from Maridi County, South Sudan[45,46], n = 774.

Supplementary References

1. Murdoch, M. E., Murdoch, I. E., Evans, J., Yahaya, H., Njepuome, N., Cousens, S., Jones, B. R. & Abiose, A. Pre-control relationship of onchocercal skin disease with onchocercal infection in Guinea Savanna, Northern Nigeria. *PLoS Negl. Trop. Dis*. **11,** e0005489 (2017).
2. Brown, L. D., Cat, T. T. & DasGupta, A. Interval estimation for a proportion. *Stat. Sci.* **16,** 101–133 (2001).
3. Hamley, J. I. D., Milton, P., Walker, M. & Basáñez, M.-G. Modelling exposure heterogeneity and density dependence in onchocerciasis using a novel individual-based transmission model, EPIONCHO-IBM: implications for elimination and data needs. *PLoS Negl. Trop. Dis.* **13,** e0007557 (2019).
4. Filipe, J. A. N., Boussinesq, M., Renz, A., Collins, R. C., Vivas-Martinez, S., Grillet, M. E., Little, M. P. & Basáñez, M.-G. Human infection patterns and heterogeneous exposure in river blindness. *Proc. Natl. Acad. Sci. U. S. A.* 2005; **102,** 15265–15270 (2005).
5. Basáñez, M.-G., Walker, M., Turner, H. C., Coffeng, L. E, de Vlas, S. J. & Stolk, W. A. River blindness: mathematical models for control and elimination. *Adv. Parasitol.* **94,** 247–341 (2016).
6. Walker, M., Stolk, W. A., Dixon, M. A., Bottomley, C., Diawara, L., Traoré, M. O., de Vlas, S. J. & Basáñez, M.-G. Modelling the elimination of river blindness using long-term epidemiological and programmatic data from Mali and Senegal. *Epidemics* **18,** 4–15 (2017).
7. Turner, H. C., Walker, M., Churcher, T. S. & Basáñez, M.-G. Modelling the impact of ivermectin on River Blindness and its burden of morbidity and mortality in African Savannah: EpiOncho projections. *Parasit. Vectors* **7,** 241 (2014).
8. Little, M. P., Breitling, L. P., Basáñez, M.-G., Alley, E. S. & Boatin, B. A. Association between microfilarial load and excess mortality in onchocerciasis: an epidemiological study. *Lancet* **363,** 1514–1521 (2004).
9. Walker, M., Little, M. P., Wagner, K. S., Soumbey-Alley, E. W., Boatin, B. A. & Basáñez, M.-G. Density-dependent mortality of the human host in onchocerciasis: relationships between microfilarial load and excess mortality. *PLoS Negl. Trop. Dis*. **6,** e1578 (2012).
10. Hamley, J. I. D., Walker, M., Coffeng, L. E., Milton, P., de Vlas, S. J., Stolk, W. A. & Basáñez, M.-G. Structural uncertainty in onchocerciasis transmission models influences the estimation of elimination thresholds and selection of age groups for seromonitoring. *J. Infect. Dis.* **221**(Suppl 5), S510–S518 (2020).
11. Dietz, K. Density-dependence in parasite transmission dynamics. *Parasitol. Today* **4,** 91–97 (1988).
12. Churcher, T. S., Filipe, J. A. N. & Basáñez, M.-G. Density dependence and the control of helminth parasites. *J. Anim. Ecol*. **75,** 1313–1320 (2006).
13. Dietz, K. The population dynamics of onchocerciasis. In Population dynamics of infectious diseases (ed. R. M. Anderson), pp. 209–241. London: Chapman & Hall (1982).

14. Dyson, L., Stolk, W. A., Farrell, S. H. & Hollingsworth, T. D. Measuring and modelling the effects of systematic non-adherence to mass drug administration. *Epidemics* **8,** 56–66 (2017).

15. Ozoh, G. A., Murdoch, M. E., Bissek, A. C., Hagan, M., Ogbuagu, K., Shamad, M., Braide, E. I., Boussinesq, M., Noma, M. M., Murdoch, I. E., Sékétéli, A. & Amazigo, U. V. The African Programme for Onchocerciasis Control: impact on onchocercal skin disease. *Trop. Med. Int. Health* **16,** 875–883 (2011).

16. Prost A. Latence parasitaire dans l'onchocercose. *Bull. World Health Organ*. **58,** 923–925 (1980).

17. Little, M. P., Basáñez, M.-G., Breitling, L. P., Boatin, B. A. & Alley, E. S. Incidence of blindness during the Onchocerciasis control programme in western Africa, 1971-2002. *J. Infect. Dis.* **189,** 1932–1941 (2004).

18. Kirkwood, B., Smith, P., Marshall, T. & Prost, A. Variations in the prevalence and intensity of microfilarial infections by age, sex, place and time in the area of the Onchocerciasis Control Programme. *Trans. R. Soc. Trop. Med. Hyg*. **77,** 857–861 (1983).

19. Coffeng, L. E. Onchocerciasis: the pre-control association between prevalence of palpable nodules and skin microfilariae - technical note and posterior draws for conversion equation. https://zenodo.org/records/13969100; https://doi.org/10.5281/zenodo.13969100 (2024).

20. Coffeng, L. E., Pion, S. D. S., O'Hanlon, S., Cousens, S., Abiose, A. O., Fischer, P. U., Remme, J. H. F., Dadzie, K. Y., Murdoch, M. E., de Vlas, S. J., Basáñez, M.-G., Stolk, W. A. & Boussinesq, M. Onchocerciasis: the pre-control association between prevalence of palpable nodules and skin microfilariae. *PLoS Negl. Trop. Dis.* **7,** e2168 (2013).

21. Behrend, M. R., Basáñez, M.-G., Hamley, J. I. D., Porco, T. C., Stolk, W. A., Walker, M., de Vlas, S. J. & NTD Modelling Consortium. Modelling for policy: the five principles of the Neglected Tropical Diseases Modelling Consortium. *PLoS Negl. Trop. Dis*. **14,** e0008033 (2020).

22. Plaisier, A. P., van Oortmarssen, G. J., Habbema. J. D., Remme, J. & Alley, E. S. ONCHOSIM: a model and computer simulation program for the transmission and control of onchocerciasis. *Comput. Methods Programs Biomed*. **31,** 43–56 (1990).

23. Coffeng, L. E., Stolk, W. A., Hoerauf, A., Habbema, D., Bakker, R., Hopkins, A. D. & de Vlas, S. J. Elimination of African onchocerciasis: modeling the impact of increasing the frequency of ivermectin mass treatment. *PLoS One* **9,** e115886 (2014).

24. Hamley, J. I. D., Walker, M., Coffeng, L. E., Milton, P., de Vlas, S. J., Stolk, W. A. & Basáñez, M.-G. Structural uncertainty in onchocerciasis transmission models influences the estimation of elimination thresholds and selection of age groups for seromonitoring. *J. Infect. Dis.* **221**(Suppl 5), S510–S518 (2020).

25. Stapley, J. N., Hamley, J. I. D., Basáñez, M.-G. & Walker, M. Modelling transmission thresholds and hypoendemic stability for onchocerciasis elimination. *PLoS Comput. Biol.* **21,** e1013026 (2025).

26. de Vos, A. S., Stolk, W. A., Coffeng, LE & de Vlas SJ. The impact of mass drug administration expansion to low onchocerciasis prevalence settings in case of connected villages. *PLoS Negl. Trop. Dis*. **15,** e0009011 (2021).

27. Vinkeles Melchers, N. V. S., Stolk, W. A., Murdoch, M. E., Pedrique, B., Kloek, M., Bakker, R., de Vlas, S. J. & Coffeng, L. E. How does onchocerciasis-related skin and eye disease in Africa depend on cumulative exposure to infection and mass treatment? *PLoS Negl. Trop. Dis*. **15,** e0009489 (2021).

28. Murdoch, M. E., Asuzu, M. C., Hagan, M., Makunde, W. H., Ngoumou, P., Ogbuagu, K. F., Okello, D., Ozoh, G. & Remme, J. Onchocerciasis: the clinical and epidemiological burden of skin disease in Africa. *Ann. Trop. Med. Parasitol*. **96,** 283–296 (2002).

29. Prost, A. & Vaugelade, J. La surmortalité des aveugles en zone de savane ouest-africaine [Excess mortality among blind persons in the West African savannah zone]. *Bull. World Health Organ*. **59,** 773–776 (1981).

30. Abiose, A., Murdoch, I., Babalola, O., Cousens, S., Liman, I., Onyema. J., Evans, J., Gregory, W. & Jones, B. Distribution and aetiology of blindness and visual impairment in mesoendemic onchocercal communities, Kaduna State, Nigeria. Kaduna Collaboration for Research on Onchocerciasis. *Br. J. Ophthalmol.* **78,** 8–13 (1994).

31. Remme, J. H. F. Global burden of onchocerciasis in 1990. World Health Organization. https://docslib.org/doc/5539825/the-global-burden-of-onchocerciasis-in-1990 (2004).

32. Remme, J., Dadzie, K.Y., Rolland, A. & Thylefors, B. Ocular onchocerciasis and intensity of infection in the community. I. West African savanna. *Trop. Med. Parasitol.* **40,** 340–347 (1989).

33. Dadzie, K. Y., Remme, J., Rolland, A. & Thylefors, B. Ocular onchocerciasis and intensity of infection in the community. II. West African rainforest foci of the vector *Simulium yahense*. *Trop. Med. Parasitol.* **40,** 348–54 (1989).

34. Dadzie, K. Y., Remme, J., Baker, R. H., Rolland, A. & Thylefors, B. Ocular onchocerciasis and intensity of infection in the community. III. West African rainforest foci of the vector *Simulium sanctipauli*. *Trop. Med. Parasitol*. **41,** 376–382 (1990).

35. Dadzie, K. Y., De Sole, G. & Remme, J. Ocular onchocerciasis and the intensity of infection in the community. IV. The degraded forest of Sierra Leone. *Trop. Med. Parasitol.* **43,** 75–79 (1992).

36. Brown, R. & Shannon, R. Prevalence, intensity and ocular manifestations of *Onchocerca volvulus* infection in Dimbelenge, Zaire. *Ann. Soc. Belg. Med. Trop*. **69,** 137–142 (1989).

37. Henry, M. C. & Maertens, K. The onchocerciasis focus at Kinsuka/Kinshasa (Republic of Zaire) in 1985. II. Parasitological and clinical aspects. *Ann. Trop. Med. Parasitol.* **84,** 493–502 (1990).

38. Whitworth, J. A., Gilbert, C. E., Mabey, D. M., Maude, G. H., Morgan, D. & Taylor, D. W. Effects of repeated doses of ivermectin on ocular onchocerciasis: community-based trial in Sierra Leone. *Lancet* **338,** 1100–1103 (1991).

39. Whitworth, J. A., Gilbert, C. E., Mabey, D. M., Morgan, D. & Foster, A. Visual loss in an onchocerciasis endemic community in Sierra Leone. *Br. J. Ophthalmol.* **77,** 30–32 (1993).

40. Kayembe, D. L., Kasonga, D. L., Kayembe, P. K., Mwanza, J.-C. K. & Boussinesq M. Profile of eye lesions and vision loss: a cross-sectional study in Lusambo, a forest-savanna area hyperendemic for onchocerciasis in the Democratic Republic of Congo. *Trop. Med. Int. Health* **8,** 83–89 (2003).

41. Stapley, J. N., Hamley, J. I. D., Walker, M., Dixon, M. A., Colebunders, R. & Basáñez, M.-G. Modelling onchocerciasis-associated epilepsy and the impact of ivermectin treatment on its prevalence and incidence. *Nat. Commun.* **15,** 6275 (2024).

42. Bhattacharyya, S., Vinkeles Melchers, N. V. S., Siewe Fodjo, J. N., Vutha, A., Coffeng, L. E., Logora, M. Y., Colebunders, R. & Stolk, W. A. Onchocerciasis-associated epilepsy in Maridi, South Sudan: modelling and exploring the impact of control measures against river blindness. *PLoS Negl. Trop. Dis*. **17,** e0011320 (2023).

43. Chesnais, C. B., Nana-Djeunga, H. C., Njamnshi, A. K., Lenou-Nanga, C. G., Boullé, C., Bissek, A. Z., Kamgno, J., Colebunders, R. & Boussinesq, M. The temporal relationship between onchocerciasis and epilepsy: a population-based cohort study. *Lancet Infect. Dis.* **18,** 1278–186 (2018).

44. Chesnais, C. B., Bizet, C., Campillo, J. T., Njamnshi, W. Y., Bopda, J., Nwane, P., Pion, S. D., Njamnshi, A. K. & Boussinesq M. A second population-based cohort study in Cameroon confirms the temporal relationship between onchocerciasis and epilepsy. *Open Forum Infect. Dis.* **7,** ofaa206 (2020).

45. Colebunders, R., Carter, J. Y., Olore, P. C., Puok, K., Bhattacharyya, S., Menon, S., Abd-Elfarag, G., Ojok, M., Ensoy-Musoro, C., Lako, R. & Logora, M. Y. High prevalence of onchocerciasis-associated epilepsy in villages in Maridi County, Republic of South Sudan: a community-based survey. *Seizure* **63,** 93–101 (2018).

46. Colebunders, R., Abd-Elfarag, G., Carter, J. Y., Olore, P. C., Puok, K., Menon, S., Siewe Fodjo, J. N., Bhattacharyya, S., Ojok, M., Lako, R. & Logora, M. Y. Clinical characteristics of onchocerciasis-associated epilepsy in villages in Maridi County, Republic of South Sudan. *Seizure* **62,** 108–115 (2018).
